# Weight Loss Trends in Women’s Wrestling and Potential Implications of Menstrual Cycle: A Narrative Review

**DOI:** 10.3390/nu18020182

**Published:** 2026-01-06

**Authors:** Andrew R. Jagim, Jennifer B. Fields, Margaret T. Jones

**Affiliations:** 1 Sports Medicine, Mayo Clinic Health System, La Crosse, WI 54601, USA; jagim.andrew@mayo.edu; 2 Exercise & Sport Science Department, University of Wisconsin—La Crosse, La Crosse, WI 54601, USA; 3 Patriot Performance Laboratory, Frank Pettrone Center for Sports Performance, George Mason University, Fairfax, VA 22030, USA; 4 Department of Nutritional Sciences, University of Connecticut, Storrs, CT 06269, USA; jennifer.fields@uconn.edu; 5 Sport, Recreation, and Tourism Management, George Mason University, Fairfax, VA 22030, USA

**Keywords:** wrestling, female wrestlers, weight loss, weight class, combat sports

## Abstract

**Background/Objectives**: Women’s wrestling is one of the fastest growing sports within the United States at the high school and collegiate level. Weight-class sports, such as wrestling, present unique challenges for female athletes, particularly in managing acute weight fluctuations associated with the menstrual cycle. However, less is known regarding how sex-specific physiology may influence weight management strategies among female wrestlers. The purpose of this review was to highlight current weight loss trends among high school and collegiate female wrestlers and summarize the physiological mechanisms driving potential menstrual-related fluid retention and subsequent changes in body mass or composition. **Methods**: A literature review was conducted to include studies evaluating weight loss trends in female wrestlers competing in the United States. Additional literature focused on fluid, body mass, and body composition changes throughout the menstrual cycle was also included. **Results**: Recent evidence has provided observational data that can be used to develop descriptive summaries of current body composition profiles and weight loss trends among female wrestlers. These data can help to quantify the typical magnitude of weight loss changes observed in female wrestlers competing at the high school and collegiate level within the United States. **Conclusions**: These findings help provide insight into the magnitude of weight loss wrestlers undergo in an effort to compete in their minimal weight class, thus informing practitioners of potential health risks and helping guide optimal weight management efforts.

## 1. Introduction

Women’s wrestling is one of the fastest-growing scholastic and collegiate sports in the United States, prompting an urgent need for sex-specific, sport-science research to support athlete health and performance [1]. Although weight-class sports have historically focused on male athletes, emerging evidence demonstrates that female wrestlers differ substantially from their male counterparts in body composition profiles, growth and maturation patterns, and physiological responses to training and weight manipulation. These differences challenge the direct application of male-based data and highlight the need for research that specifically accounts for the unique physiological and regulatory considerations relevant to females [1,2].

A central component of weight-class safety policy for wrestling in the United States is the use of the Minimum Wrestling Weight (MWW) program, which establishes the lowest competitive weight class an athlete may safely achieve based on the athlete’s current body weight, body composition, and projected weight using minimum body-fat thresholds [1,2]. Recent large-scale datasets describing MWW distribution and body composition characteristics among high school and collegiate female wrestlers have provided much-needed foundational information [3,4]. However, these descriptive reports do not fully consider how female-specific physiology, particularly the menstrual cycle, may influence body mass, hydration status, and weight-cutting outcomes, nor how these fluctuations interact with regulatory systems such as MWW-based descent plans. This represents a critical research gap, as female athletes experience hormone-driven changes in total body water, fluid-regulating hormones, and thermoregulation across the menstrual cycle. Such fluctuations have been purported to alter scale weight by ~0.5–1.0 kg, independent of fat or lean tissue, with potentially meaningful implications for weight-class sports. For wrestlers operating near MWW boundaries, even small changes in water retention can affect compliance with descent plans, the accuracy of body-composition assessments, the effectiveness of hydration-based weight-cutting strategies, and ultimately the ability to make weight safely. Despite this, the existing literature on women’s wrestling rarely integrates menstrual physiology into discussions of weight-management practices, leaving coaches and practitioners without clear, evidence-based guidance.

Further complicating this issue is the widespread use of acute (~24–72 h prior to weigh-in) weight-loss strategies, including dehydration, caloric restriction, and increased training load, which may interact with menstrual-cycle physiology in ways that increase risk [5,6,7,8]. For instance, if weigh-ins coincide with periods of elevated fluid retention (e.g., late luteal phase), athletes may engage in excessive dehydration tactics to compensate for transient, hormonally driven mass fluctuations. These practices could compound health risks and inadvertently undermine performance, especially given the already limited safe weight-loss capacity for many female wrestlers competing near their MWW.

Wrestling, which often requires athletes to weigh in at specific times and adhere to strict weight limits, amplifies the need to understand and anticipate these variations [1,9]. Taken together, these factors underscore the need for an integrated review that synthesizes current data on body composition and MWW trends in female wrestlers, explains the physiological mechanisms underlying menstrual-cycle-related fluctuations in body water and body mass, and translates this information into practical, sex-specific recommendations for weight-class selection, hydration management, and timing of assessments such as weigh-ins and body-composition testing.

Therefore, the purpose of this narrative review is to quantify the magnitude of weight loss exhibited by female wrestlers from the time of weight certification to their lowest competition weight during the season. Additionally, we aimed to bridge the gap between regulatory guidelines (i.e., MWW), current weight loss practices in female wrestlers, female physiology (i.e., menstrual-cycle effects on hydration and body mass), and applied weight-management strategies in women’s wrestling. By integrating these concepts, this review will provide coaches, clinicians, and sports performance staff with a cohesive framework for supporting the health, safety, and performance of female wrestlers.

## 2. Materials and Methods

### Search Strategy

A narrative review was conducted over 3 months, spanning from August 2025 to November 2025. The following online databases were used to search relevant literature for inclusion: PubMed, Google Scholar, Scopus, Web of Science, and EBSOHOST. The primary search was focused on articles that evaluated weight loss trends throughout the season in female wrestlers competing in the United States. Wrestlers competing at both the high school and collegiate levels were included. The following keyword combinations and search string was used: (wrestling OR “weight-class sport*” OR “combat sport*”) AND (“minimum wrestling weight” OR MWW OR “weight certification” OR “body composition”) AND (“weight loss” OR “weight cutting” OR dehydration OR “rapid weight loss”).

The keyword set was intentionally designed to capture the primary domains relevant to weight-management research in women’s wrestling within the United States (US). The search strategy employed multiple combinations of these terms to maximize identification of pertinent studies. Eligibility criteria were restricted to observational investigations, case studies, and case reports involving female wrestlers. Articles that included non-wrestling athletes, non-US wrestlers, failed to quantify pre-season and within season body weight values, and included male wresters were excluded. Studies across any time period were eligible to be included in the review.

## 3. Results

### 3.1. Study Characteristics

**Figure 1:**
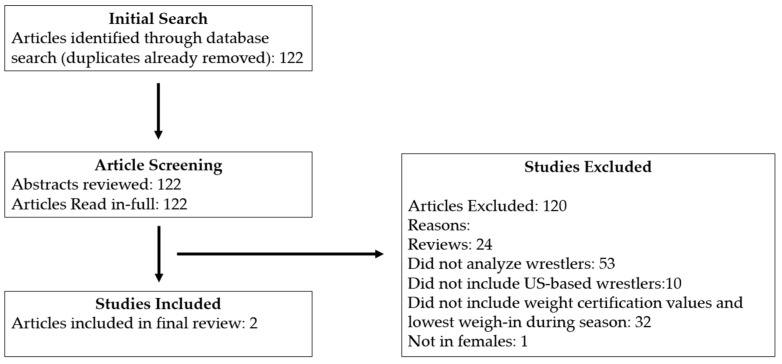
Flow diagram depicting the article search and screening process.

The initial search identified 122 studies. Of these articles, 120 articles were excluded as they did not meet inclusion criteria. The review and exclusion process is outlined in Figure 1.

Two studies were identified that met the inclusion criteria for protocols that quantified body weight changes in female wrestlers competing in the US (Table 1). One study retrospectively examined 1683 collegiate women wrestlers [4] and another retrospectively examined 33,321 high school female wrestlers [3], both of which represented multiple programs across the United States.

For each study, athletes underwent body composition assessment via skinfold (collegiate) or bioelectrical impedance analysis (high school only) to determine fat mass (FM), fat-free mass (FFM), and body fat percentage (BF%) in accordance with the sport governing bodies at each respective level of competition. MWW was obtained from the National Wrestling Coaches Association (NWCA) weight certification protocol, which applies a minimum BF% threshold of 12% for female wrestlers (Table 2). Using this cutoff, each athlete’s projected safe competitive weight was calculated and aligned with the closest official collegiate women’s wrestling weight class. Comparisons were made across weight classes to identify differences in BF%, FFM, and MWW relative to current body mass.

### 3.2. Body Weight Trends in Women’s Wrestling

On average, MWW was 7.7% lower than actual body mass at the time of weight certification for collegiate wrestlers and 7.4% lower for high school wrestlers. The average allowable drop in weight class was approximately one division lower than the athlete’s current class; however, this varied considerably, with some athletes already competing at or near their MWW. For college wrestlers, only 354 out of 1579 (22.4%) wrestlers competed in their lowest allowable weight class. Of these 354 wrestlers, the mean BF% was 21.3 ± 5.2% at weight certification, with only 17 athletes being at or below 12% body fat and an average weight loss of 5.0 ± 3.9 kg from the time of weight certification. High school wrestlers were, on average, 1–2 weight classes above their MWW competition class at the time of weight certification.

### 3.3. Body Fat Percentage in Women’s Wrestling

For each study, percentile rankings were established for collegiate and high school wrestlers, helping to provide context in regard to specific ranges for BF% and MWW for each respective level of competition (Table 3). At the high school level, the median ± interquartile range for BF% was 28.3 ± 9.2% [3], slighter higher than the 27.4 ± 10.22% [4] observed for BF% at the collegiate level. Over 95% of the wrestlers included in both studies exhibited BF% values above 17%. For the collegiate wrestlers, heavier weight classes displayed greater absolute FFM and FM, but BF% varied less dramatically across classes. Light and middle weight classes generally had BF% values in the low 20s, while the highest classes averaged slightly above this range. At the high school level, absolute FFM increased with higher weight classes, as expected. Lighter weight classes displayed lower absolute FM but not drastically lower BF% compared to middle weight classes. The heaviest classes had greater FM and BF%, although many still fell within healthy adolescent norms.

## 4. Discussion

### 4.1. Weight Loss Trends and Body Composition Profiles

The primary aim of the current review was to evaluate common weight loss trends and body composition profiles in female wrestlers competing in the United States. The data underscores the value of MWW assessments in preventing unsafe weight loss practices. For athletes significantly above their MWW, gradual weight reduction strategies may be feasible. Conversely, for those already at or near MWW, emphasis should shift toward maintaining strength and power in their current weight class rather than pursuing further weight loss. Attempting to drop below MWW risks reductions in lean mass, impaired recovery, and potential hormonal disturbances [1,9]. The average ~7% difference between pre-season body weight and MWW provides a reasonable upper bound for in-season weight loss goals in female wrestlers. Given the health risks of aggressive cutting, especially in still-developing female athletes, weight reduction should occur gradually, ideally during the preseason or offseason, with adequate nutritional support [1]. Moreover, both the National Federation of High School Sports and the NCAA restrict weekly weight loss to no more than 1.5% of body weight per week.

The average BF% of ~27–28% aligns with values reported in other female collision and combat sports, though slightly higher than those seen in elite endurance athletes [10,11,12]. Importantly, these levels fall within healthy ranges for female athletes, highlighting that excessively low BF% is neither necessary nor common in today’s women’s wrestling. Coaches should recognize that optimal performance may occur at higher BF% values than in men’s wrestling, given sex-based physiological differences in essential fat levels and hormonal function. For high school wrestlers, the average BF% of ~28% also aligns with healthy ranges for active adolescent females [13,14]. Importantly, during adolescence, body composition is influenced by ongoing growth and maturation, including changes in bone mineral content, muscle mass, and essential fat levels [15,16]. Coaches must account for these developmental processes when interpreting BF% values and determining competitive weight classes. Unlike adult athletes, high school athletes may experience seasonal or yearly increases in FFM simply through natural growth, independent of targeted training. Rapid or excessive weight loss during these formative years may impair growth potential, disrupt menstrual function, and increase injury risk [7,17].

These findings can inform pre-season weight class selection, along with nutritional and training-related periodization strategies throughout the season. For athletes already at or near MWW, performance optimization should take precedence over weight loss [9,18]. This approach may help prevent fatigue, impair recovery, and decrease strength that can result from energy restriction. Athletes with larger gaps between pre-season body weight and MWW may target gradual off-season changes, while those near MWW should focus on technical, tactical, and conditioning improvements without major body composition shifts [1,3]. Because high school athletes may gain height and FFM during the school year, targeting a weight class significantly below their natural growth trajectory is both unrealistic and potentially harmful [1,3,9]. Coaches should consider an athlete’s historical growth patterns and biological maturation stage when setting weight goals [3,9,18].

Periodic reassessment of body weight and adherence to the descent plan trajectory throughout the season is recommended, especially for underclassmen who may experience substantial changes in size and composition. Clear communication about safe weight management is essential. Many adolescent wrestlers and their families may be unaware of the physiological minimums embedded in MWW calculations and/or why a descent plan is set in place to ensure safe and stepwise reductions in body weight. Educating athletes on the importance of lean mass preservation and the risks of extreme or rapid weight loss can help promote healthier behaviors and a sustainable competitive career.

### 4.2. Female-Specific Physiology and Implications for Sport

Optimizing performance and recovery in female athletes requires consideration of sex-specific physiology, including the influence of the menstrual cycle on hydration and weight management [19]. An often-overlooked factor is the cyclical regulation of fluid balance, whereby fluctuations in estrogen and progesterone alter fluid-regulating hormones such as aldosterone and antidiuretic hormone, potentially affecting total body water [20,21,22]. These hormonal shifts may confound assessments of body mass, fat mass, fat-free mass, and performance, leading to misinterpretation of changes that are transient and hormonally driven rather than training-related [23,24]. Accordingly, understanding menstrual-cycle-related fluid regulation is important for interpreting body composition data and informing weight management strategies in female wrestlers. It is important to note that these interpretations represent contextualized applications of established physiological mechanisms, rather than direct causal evidence in female wrestlers.

### 4.3. Menstrual Cycle Overview

The menstrual cycle (~28 days) is characterized by fluctuations in estrogen and progesterone that influence fluid balance, thermoregulation, and cardiovascular function, with potential implications for weight management in female athletes [19,22], as outlined in Table 4.

**Figure 2:**
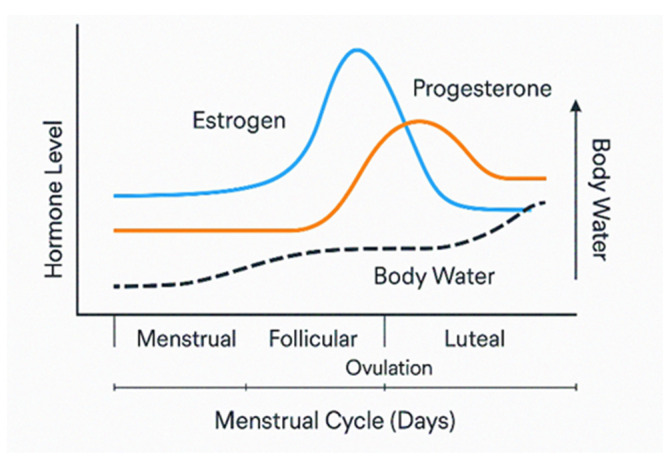
Hormone and Body Water Changes Across the Menstrual Cycle.

Interactions between these hormones and the renin–angiotensin–aldosterone system may promote transient sodium and water retention, particularly around ovulation and during the luteal phase [20,21,22]. Specifically, estrogen increases angiotensinogen production, stimulating RAAS, which can promote sodium and water retention, particularly around ovulation [21]. Additionally, this can enhance vascular permeability, potentially contributing to mild edema. Progesterone has complex effects, contributing to plasma volume expansion and bloating while also promoting natriuresis through non-genomic pathways [21], as seen in Figure 2. Importantly, evidence regarding the magnitude and consistency of menstrual-cycle-related fluid shifts is mixed, suggesting substantial individual variability and the involvement of additional regulatory mechanisms.

### 4.4. Total Body Water and Body Weight Fluctuations Across the Menstrual Cycle

Menstrual-cycle-related hormonal fluctuations may contribute to short-term changes in total body water (TBW) and body mass, which can be particularly relevant in weight-category sports where small weight differences are consequential [21] (Table 5).

Some studies report transient fluid-driven weight increases of ~0.5–1.0 kg during the late luteal phase that typically resolve with the onset of menstruation; however, findings across active women are inconsistent, with several investigations reporting minimal or no measurable changes in body mass, TBW, or body-composition compartments across the cycle [23,24,25,26,27,28,29]. This variability suggests substantial inter-individual differences and the influence of additional physiological and lifestyle factors.

From an applied perspective, even modest fluid shifts may complicate the interpretation of body-composition assessments and short-term weight changes in female wrestlers. Techniques reliant on body-water assumptions, such as bioelectrical impedance analysis, may be particularly sensitive to these fluctuations, potentially inflating estimates of fat mass or lean mass depending on extracellular water distribution [28,30,31,32,33,34]. Although absolute changes in TBW are often small, they may contribute to bloating, perceived discomfort, or thermoregulatory strain in some athletes, which could influence performance and weight-making strategies [35]. Hormonal contraceptive use may further modify baseline TBW by attenuating natural hormonal variability [36,37].

Collectively, the inconsistent nature of menstrual-cycle-related changes in body water and body mass underscores the importance of cautious interpretation of short-term weight fluctuations. Practitioners working with female wrestlers should avoid attributing small increases in body mass solely to changes in body composition or training adherence and instead consider menstrual-cycle phase and individual variability when monitoring weight, hydration status, and readiness to compete.

## 5. Relevance to Female Wrestlers

Though direct sport performance ability may not be significantly impaired by BM increases of 1–2%, subjective feelings of heaviness or bloating, especially in weight-category sports such as wrestling, can impact psychological readiness [35,38]. Most female athletes indicate that symptom prevalence and severity tend to be the most common during the early days of menstruation [38]. Coaches, dietitians, and athletic trainers should be cautious when setting weight targets or evaluating progress during the luteal phase. In the context of weight-class competition, female wrestlers face additional challenges because of the hormonal and subsequent physiological changes occurring throughout the menstrual cycle. For example, they may experience weight gain due to fluid retention may occur despite energy restriction or training adherence. Traditional dehydration protocols (e.g., saunas, water loading) may be less effective when baseline water retention is elevated [39]. Last-minute acute weight loss strategies may lead to greater cardiovascular strain, impaired thermoregulation, or heat-related illness [1,39].

During the late luteal phase, women can retain 0.5–1.0 kg (1–2.5 lbs.) of additional water weight, although there tends to be a high degree of individual variability. This could have the potential to mimic weight/fat gain and ultimately mislead athletes. Athletes should be informed that this may disrupt tracking of body composition or weight loss trajectories (“descents”). Subsequently, this may cause unexpected weigh-in failures, increased pressure for excessive last-minute dehydration strategies, thereby increasing health risks, and misinterpretation of “failure” in diet or training. In summary of the information presented in the current review, along with additional research on weight-category sports and female athletes, and expert opinion [1,9,18,38,39,40,41,42,43,44,45,46,47], practical strategies are proposed in Table 6 to help coaches and athletes optimize performance and health throughout a wrestling season in an effort to appropriately manage their weight and performance throughout the year. These recommendations should be individualized, applied conservatively, and re-evaluated as sport-specific research emerges.

### Limitations and Directions for Future Research

Several limitations should be acknowledged when interpreting the findings of this narrative review. First, the current evidence base examining weight-loss patterns, body-composition trends, and menstrual-cycle-related fluid shifts in female wrestlers remains exceedingly narrow. Only two studies to date have quantified in-season or pre-season weight-change patterns in U.S. female wrestlers, and both relied on retrospective analyses of large administrative datasets rather than prospective, mechanistic, or experimental designs. As a result, the generalizability of available data is limited, and important sport-specific questions, such as how female wrestlers respond to acute weight-cutting practices across menstrual phases, remain largely unanswered. Additionally, different methods of body composition assessment were utilized (BIA and Skinfold) across the two studies. However, previous research [3] noted that while a difference in mean BF% values between BIA (28.4 ± 6.8%) and SKF (29.4 ± 7.6%) was observed, the difference of 1% between the methods was found to result in a trivial effect size and within the error of the methods [48].

Second, much of the broader literature used to contextualize potential physiological mechanisms surrounding fluid balance and menstrual-cycle-related changes is derived from non-athletic samples or from endurance, team sport, or recreationally active female populations. Because wrestling uniquely combines weight-class constraints, high training loads, rapid recovery periods, and frequent weigh-ins, extrapolating findings from these populations may not fully capture the sport-specific physiological demands or behavioral patterns of wrestlers. The absence of controlled studies conducted directly in competitive female wrestlers represents a critical gap, particularly given the sport’s increasing participation and the unique risk profile associated with rapid weight management.

Third, substantial methodological heterogeneity across the literature limits the ability to draw firm conclusions regarding weight and fluid shifts across the menstrual cycle in female athletes. Studies included in the menstrual-cycle domain varied widely in assessment tools (e.g., bioelectrical impedance analysis, DXA, skinfolds), menstrual-cycle tracking methods (calendar-based, hormone-confirmed, or unverified self-report), hydration standardization procedures, training-status control, and timing of measurements across the cycle. These inconsistencies complicate interpretation, as small changes in body water, which is central to weight-class sports, may subsequently reflect methodological artifacts rather than true physiological fluctuations. Additionally, the retrospective wrestling datasets incorporated different body-composition technologies between levels (skinfolds vs. BIA), which may introduce measurement variability when comparing outcomes across populations.

Future work should prioritize prospective, sport-specific research in competitive female wrestlers, including carefully controlled studies across menstrual phases; mechanistic investigations into hydration, thermoregulation, and acute weight-cut responses. Additionally, more work is needed to validate body-composition and hydration-assessment tools within this population. Such efforts are needed to advance evidence-based guidelines for safe and effective weight management in women’s wrestling. Despite growing interest in female physiology in sport, much of the evidence surrounding menstrual-cycle-related weight changes is mostly observational, based on small sample sizes, and lacking in elite combat sport populations [1]. Further work is needed to quantify the exact fluid shifts in elite wrestlers, compare cutting responses across phases, and develop sex-specific hydration and nutrition strategies.

## 6. Conclusions

In summary, MWW remains a central and foundational component of safe weight-class determination for female wrestlers, particularly given the proportion of athletes competing near their lower allowable limits. Additionally, MWW provides a health-anchored baseline that constrains how much weight loss is both permissible and advisable, and therefore represents an essential consideration for coaches and practitioners working with this population. This can also help with determining safe competitive weight classes and guiding weight management strategies.

Currently, collegiate women’s wrestlers display healthy and sport-appropriate body composition profiles, with BF% values averaging in the mid-20s and considerable inter-individual variation. However, given the limited number of studies on female wrestlers, conclusions should be interpreted cautiously. Practitioners should resist applying male wrestling norms to female wrestlers and instead adopt sex-specific benchmarks that account for physiological differences. By leveraging objective MWW assessments, coaches and sports health professionals can help athletes compete at weights that maximize performance while safeguarding long-term health.

Menstrual-cycle-related fluid retention may influence short-term body mass fluctuations, but the magnitude, direction, and consistency of these changes vary considerably across individuals and across studies. While some women may experience transient increases in body mass (~0.5–1.0 kg) in the late luteal phase, the current body of literature is mixed, with several studies reporting minimal or no measurable fluid shifts. As such, cycle-related water retention may complicate weight-cutting strategies for some athletes but should not be interpreted as a universal or deterministic physiological barrier. Understanding and anticipating the hormonal shifts is crucial for practitioners seeking to optimize athlete health, safety, and performance. With proper education, monitoring, and individualized plans, strength and conditioning coaches can ensure that female wrestlers manage weight fluctuations safely while preserving performance potential.

## Figures and Tables

**Table 1:** Summary of Study Characteristics.

Characteristic	Jagim et al. [4]	Jagim et al. [3]
Sample	1683	33,321
Year	2022–2023	2022–2023
Sex	Female	Female
Level of Competition	College	High School
Body Fat %	27.4 ± 10.2	28.3 ± 9.2
Method of Body Composition	Skinfold	Skinfold; BIA

BIA = bioelectrical impedance analysis.

**Table 2:** Summary of Pre-Season Body Composition Parameters in Female Wrestlers.

Body Composition Parameter	College	High School
Body Mass (kg)	66.8 ± 13.9	63.4 ± 14.0
Body Fat Percentage (%)	27.4 ± 10.2	28.3 ± 9.2
Fat-free Mass (kg)	55.2 ± 6.6	45.5 ± 5.3
Minimum Wrestling Weight (kg)	54.1 ± 8.4	52.2 ± 10.1
Current Weight—MWW (kg)	12.7 ± 7.8	11.2 ± 6.4

MWW = minimal wrestling weight; kg = kilograms; % = percentage. Adapted from Jagim et al. [3] and Jagim et al. [4].

**Table 3:** Percentiles for Body Fat Percentage and Minimum Wrestling Weight.

	5th	25th	50th	75th	95th
Body Fat (%)					
College	17.2%	22.6%	27.4%	32.9%	43.2%
High School	18.9%	23.9%	28.3%	33.7%	44.0%
Minimal Wrestling Weight (kg)					
College	42.4	48.0	52.9	58.9	69.4
High School	40.4	46.0	51.0	58.0	74.8

kg = kilograms; % = percentage. Adapted from Jagim et al. [3] and Jagim et al. [4].

**Table 4:** Summary of Menstrual Cycle and Corresponding Hormonal and Physiological Responses.

Phase	Days	Hormonal Profile	Reproductive Changes
Menstrual	1–5	Low estrogen and progesterone	Shedding of the uterine lining
Follicular	6–14	Rising estrogen, low progesterone	Follicle development
Ovulation	~14	Estrogen peaks, LH/FSH surge	Release of the oocyte
Luteal	15–28	High progesterone, moderate estrogen	Formation of the corpus luteum

**Table 5:** Hormonal Effects on Body Water Retention.

Hormonal	Body Water Changes
Estrogen	Increases angiotensinogen production → stimulates the renin–angiotensin–aldosterone system (RAAS). Promotes sodium and water retention. May lead to mild extracellular water (ECW) increases, particularly around ovulation.
Progesterone	Has aldosterone-like effects, which can increase sodium retention. However, also acts as a natriuretic via progesterone receptors → potentially offsets water retention. It can cause plasma volume expansion, bloating, and increased thirst in the luteal phase.

**Table 6:** A summary of practical considerations for coaches and practitioners to support women wrestlers throughout the season.

Goals	Objective	Practical Strategies
Cycle Tracking	Encourage athletes to use tools (e.g., Clue, FitrWoman) to track:	Note cycle length and phases. Pay attention to common patterns with symptoms (bloating, fatigue) and weight fluctuations. If competition/weigh in is unavoidable during Luteal Phase (~days 24–28), allow more days to gradually reduce body mass. Moderate sodium intake during luteal phase. Emphasize consistent hydration to prevent reactive fluid shifts.
Integrate MWW Testing into Preseason Assessments	Body Composition Testing	Avoid scheduling peak weight certification testing or even weigh-ins during the late luteal phase (~days 24–28) to prevent inflated BF% estimates. If possible, establish each athlete’s safe lower limit before the competition season begins to allow for time to meet the descent plan. Use this data to guide individualized plans and help guide realistic, health-centered weight class assignments, while minimizing the need for aggressive rapid weight loss protocols.
Educating Healthy BF% Ranges	Prioritize Growth and Long-Term Development Preserve Lean Mass During Weight Loss:	Recognize that adolescent wrestlers are still maturing physically and may increase lean mass naturally over the season or school year, therefore they likely need to go a weight class from year to year. If an athlete’s BF% is below 12%, adjustments should be made immediately to increase weight to avoid health risks. Reinforce that women’s optimal performance often occurs at higher BF% levels than men’s, and that extremely low values can be detrimental. Implement resistance training and protein intakes of ~1.6–2.2 g/kg body mass to mitigate reductions in lean body mass during periods of intentional energy restriction and weight loss.
Avoid Rapid Weight Loss	Develop a week-to-week strategy for weight management Weigh-in Timing Hydration Strategy Adjustments:	Limit reductions to ≤1.5% of body mass per week and ensure adequate energy and protein intake (≥1.6 g/kg/day) to preserve lean tissue throughout a stepwise body weight reduction approach. Minimize rapid weight loss of >5% in the week leading up to weigh-ins for competition to minimize the risks of performance reductions and dehydration-related health effects. Develop re-fueling and re-hydration strategy post-weigh in to optimize recovery timeline prior to competition.
Monitor Throughout the Season	Focus on Performance Metrics Over Scale Weight Educating Parents and Athletes	Encourage athletes near MWW to concentrate on skill development, conditioning, and recovery rather than weight manipulation. Reassess body composition and weight trajectories to ensure strategies remain aligned with health and performance goals. Provide age-appropriate guidance on nutrition, hydration, and recovery to foster a supportive environment for safe weight management. Educating athletes that gain weight during certain phases of the menstrual cycle is likely temporary and not reflective of fat gain or fitness loss.

## Data Availability

No new data were created or analyzed in this study.

## Abbreviations

The following abbreviations are used in this manuscript:

MWW	Minimal wrestling weight
BF%	Body fat percentage
FFM	Fat-free mass
FM	Fat mass

## References

[1] Jagim A., Woodroffe L.M., Sadoski J., Horswill C., Bloomfield S., Oppliger R. (2024). Contemporary Issues: Health & Safety of Female Wrestlers. Med. Sci. Sports Exerc..

[2] Oppliger R.A., Harms R.D., Herrmann D.E., Streich C.M., Clark R.R. (1995). The Wisconsin wrestling minimum weight project: A model for weight control among high school wrestlers. Med. Sci. Sports Exerc..

[3] Jagim A.R., Horswill C.A., Dobbs W.C., Twohey E.E., Tinsley G.M., Fields J.B., Jones M.T. (2025). Minimum Wrestling Weight for High School Girls Wrestling: Time to Revisit Minimal Body Fat Percent. J. Strength Cond. Res..

[4] Jagim A.R., Tinsley G.M., Oppliger R.A., Horswill C.A., Dobbs W.C., Fields J.B., Cushard C., Rademacher P.D., Jones M.T. (2024). Collegiate women’s wrestling body fat percentage and minimum wrestling weight values: Time for revisiting minimal body fat percent?. J. Int. Soc. Sports Nutr..

[5] Jones L.K., Meyer N.L., Gibson J.C. (2014). Weight Management Practices of 2012 Olympians in Combat Sports. Int. J. Wrestl. Sci..

[6] Kasper A.M., Crighton B., Langan-Evans C., Riley P., Sharma A., Close G.L., Morton J.P. (2019). Case Study: Extreme Weight Making Causes Relative Energy Deficiency, Dehydration, and Acute Kidney Injury in a Male Mixed Martial Arts Athlete. Int. J. Sport Nutr. Exerc. Metab..

[7] Lakicevic N., Reale R., D’Antona G., Kondo E., Sagayama H., Bianco A., Drid P. (2022). Disturbing Weight Cutting Behaviors in Young Combat Sports Athletes: A Cause for Concern. Front. Nutr..

[8] Lebron M., Stout J.R., Fukuda D.H. (2024). Physiological Perturbations in Combat Sports: Weight Cycling and Metabolic Function—A Narrative Review. Metabolites.

[9] Burke L.M., Slater G.J., Matthews J.J., Langan-Evans C., Horswill C.A. (2021). ACSM Expert Consensus Statement on Weight Loss in Weight-Category Sports. Curr. Sports Med. Rep..

[10] Giovanelli L., Biganzoli G., Spataro A., Malacarne M., Bernardelli G., Spada R., Pagani M., Biganzoli E., Lucini D. (2023). Body composition assessment in a large cohort of Olympic athletes with different training loads: Possible reference values for fat mass and fat-free mass domains. Acta Diabetol..

[11] Fields J.B., Metoyer C.J., Casey J.C., Esco M.R., Jagim A.R., Jones M.T. (2018). Comparison of Body Composition Variables Across a Large Sample of National Collegiate Athletic Association Women Athletes From 6 Competitive Sports. J. Strength Cond. Res..

[12] Magee M.K., Fields J.B., Jagim A.R., Jones M.T. (2023). Fat-Free Mass Index in a Large Sample of National Collegiate Athletic Association Men and Women Athletes From a Variety of Sports. J. Strength Cond. Res..

[13] Lohman T.G., Ring K., Schmitz K.H., Treuth M.S., Loftin M., Yang S., Sothern M., Going S. (2006). Associations of body size and composition with physical activity in adolescent girls. Med. Sci. Sports Exerc..

[14] Mateo-Orcajada A., Vaquero-Cristobal R., Esparza-Ros F., Abenza-Cano L. (2022). Physical, Psychological, and Body Composition Differences between Active and Sedentary Adolescents According to the “Fat but Fit” Paradigm. Int. J. Environ. Res. Public Health.

[15] Siervogel R.M., Demerath E.W., Schubert C., Remsberg K.E., Chumlea W.C., Sun S., Czerwinski S.A., Towne B. (2003). Puberty and body composition. Horm. Res..

[16] Loomba-Albrecht L.A., Styne D.M. (2009). Effect of puberty on body composition. Curr. Opin. Endocrinol. Diabetes Obes..

[17] Brown K.A., Dewoolkar A.V., Baker N., Dodich C. (2017). The female athlete triad: Special considerations for adolescent female athletes. Transl. Pediatr..

[18] Horswill C.A., Roedeshimer A.E. (2022). Rethinking the 12% Body-Fat Minimum for Female Wrestlers. Curr. Sports Med. Rep..

[19] Constantini N.W., Dubnov G., Lebrun C.M. (2005). The menstrual cycle and sport performance. Clin. Sports Med..

[20] Giersch G.E.W., Charkoudian N., Stearns R.L., Casa D.J. (2020). Fluid Balance and Hydration Considerations for Women: Review and Future Directions. Sports Med..

[21] Stachenfeld N.S., DiPietro L., Kokoszka C.A., Silva C., Keefe D.L., Nadel E.R. (1999). Physiological variability of fluid-regulation hormones in young women. J. Appl. Physiol..

[22] Sherman B.M., Korenman S.G. (1975). Hormonal characteristics of the human menstrual cycle throughout reproductive life. J. Clin. Investig..

[23] Kanellakis S., Skoufas E., Simitsopoulou E., Migdanis A., Migdanis I., Prelorentzou T., Louka A., Moschonis G., Bountouvi E., Androutsos O. (2023). Changes in body weight and body composition during the menstrual cycle. Am. J. Hum. Biol..

[24] Meignie A., Duclos M., Carling C., Orhant E., Provost P., Toussaint J.F., Antero J. (2021). The Effects of Menstrual Cycle Phase on Elite Athlete Performance: A Critical and Systematic Review. Front. Physiol..

[25] Kosar S.N., Guzel Y., Kose M.G., Kin Isler A., Hazir T. (2022). Whole and segmental body composition changes during mid-follicular and mid-luteal phases of the menstrual cycle in recreationally active young women. Ann. Hum. Biol..

[26] Rael B., Romero-Parra N., Alfaro-Magallanes V.M., Barba-Moreno L., Cupeiro R., Janse de Jonge X., Peinado A.B., Iron F.S.G. (2021). Body Composition Over the Menstrual and Oral Contraceptive Cycle in Trained Females. Int. J. Sports Physiol. Perform..

[27] Ong J.N., Ducker K.J., Furzer B.J., Dymock M., Landers G.J. (2022). Measures of body composition via Dual-energy X-ray absorptiometry, ultrasound and skinfolds are not impacted by the menstrual cycle in active eumenorrheic females. J. Sci. Med. Sport.

[28] Moore S.R., Gordon A.N., Cabre H.E., Hackney A.C., Smith-Ryan A.E. (2023). A Randomized Controlled Trial of Changes in Fluid Distribution across Menstrual Phases with Creatine Supplementation. Nutrients.

[29] Bisson D.L., Dunster G.D., O’Hare J.P., Hampton D., Penney M.D. (1992). Renal sodium retention does not occur during the luteal phase of the menstrual cycle in normal women. Br. J. Obs. Gynaecol..

[30] Hewitt M.J., Going S.B., Williams D.P., Lohman T.G. (1993). Hydration of the fat-free body mass in children and adults: Implications for body composition assessment. Am. J. Physiol..

[31] Matias C.N., Santos D.A., Judice P.B., Magalhaes J.P., Minderico C.S., Fields D.A., Lukaski H.C., Sardinha L.B., Silva A.M. (2016). Estimation of total body water and extracellular water with bioimpedance in athletes: A need for athlete-specific prediction models. Clin. Nutr..

[32] Pialoux V., Mischler I., Mounier R., Gachon P., Ritz P., Coudert J., Fellmann N. (2004). Effect of equilibrated hydration changes on total body water estimates by bioelectrical impedance analysis. Br. J. Nutr..

[33] O’Brien C., Young A.J., Sawka M.N. (2002). Bioelectrical impedance to estimate changes in hydration status. Int. J. Sports Med..

[34] Ugras S. (2020). Evaluating of altered hydration status on effectiveness of body composition analysis using bioelectric impedance analysis. Libyan J. Med..

[35] Taim B.C., Lye J., Suppiah H.T., Chan T.W., Chia M., Clarke A. (2024). Menstrual cycle characteristics, perceived impact on performance, and barriers to communication: Perspectives of high-performance adolescent athletes in Singapore. Scand. J. Med. Sci. Sports.

[36] Stachenfeld N.S., Taylor H.S. (2004). Effects of estrogen and progesterone administration on extracellular fluid. J. Appl. Physiol..

[37] Stachenfeld N.S. (2008). Sex hormone effects on body fluid regulation. Exerc. Sport Sci. Rev..

[38] Taim B.C., Ó Catháin C., Renard M., Elliott-Sale K.J., Madigan S., Ni Cheilleachair N. (2023). The Prevalence of Menstrual Cycle Disorders and Menstrual Cycle-Related Symptoms in Female Athletes: A Systematic Literature Review. Sports Med..

[39] Reale R., Slater G., Burke L.M. (2017). Acute-Weight-Loss Strategies for Combat Sports and Applications to Olympic Success. Int. J. Sports Physiol. Perform..

[40] Barley O.R., Chapman D.W., Abbiss C.R. (2019). The Current State of Weight-Cutting in Combat Sports-Weight-Cutting in Combat Sports. Sports.

[41] Peacock C.A., French D., Sanders G.J., Ricci A., Stull C., Antonio J. (2022). Weight Loss and Competition Weight in Ultimate Fighting Championship (UFC) Athletes. J. Funct. Morphol. Kinesiol..

[42] Reale R., Slater G., Burke L.M. (2017). Individualised dietary strategies for Olympic combat sports: Acute weight loss, recovery and competition nutrition. Eur. J. Sport Sci..

[43] Reale R., Slater G., Burke L.M. (2018). Weight management Practices of Australian Olympic Combat Sport Athletes. Int. J. Sports Physiol. Perform..

[44] Tipton C.M., Oppliger R.A. (1993). Nutritional and fitness considerations for competitive wrestlers. World Rev. Nutr. Diet..

[45] Horswill C.A., Scott J.R., Dick R.W., Hayes J. (1994). Influence of rapid weight gain after the weigh-in on success in collegiate wrestlers. Med. Sci. Sports Exerc..

[46] Matthews J.J., Stanhope E.N., Godwin M.S., Holmes M.E.J., Artioli G.G. (2019). The Magnitude of Rapid Weight Loss and Rapid Weight Gain in Combat Sport Athletes Preparing for Competition: A Systematic Review. Int. J. Sport Nutr. Exerc. Metab..

[47] Mauricio C.A., Merino P., Merlo R., Vargas J.J.N., Chavez J.A.R., Perez D.V., Aedo-Munoz E.A., Slimani M., Brito C.J., Bragazzi N.L. (2022). Rapid Weight Loss of Up to Five Percent of the Body Mass in Less Than 7 Days Does Not Affect Physical Performance in Official Olympic Combat Athletes With Weight Classes: A Systematic Review With Meta-Analysis. Front. Physiol..

[48] Lohman T.G. (1981). Skinfolds and body density and their relation to body fatness: A review. Hum. Biol..

